# Regulation of Circadian Genes Nr1d1 and Nr1d2 in Sex-Different Manners during Liver Aging

**DOI:** 10.3390/ijms231710032

**Published:** 2022-09-02

**Authors:** Sang Gyun Noh, Hee Jin Jung, Seungwoo Kim, Radha Arulkumar, Dae Hyun Kim, Daeui Park, Hae Young Chung

**Affiliations:** 1 Interdisciplinary Research Program of Bioinformatics and Longevity Science, Pusan National University, 2, Busandaehak-ro 63beon-gil, Geumjeong-gu, Busan 46241, Korea; 2 Department of Pharmacy, College of Pharmacy, Pusan National University, 2, Busandaehak-ro 63beon-gil, Geumjeong-gu, Busan 46241, Korea; 3 Department of Predictive Toxicology, Korea Institute of Toxicology, 141, Gajeong-ro, Yuseong-gu, Daejeon 34114, Korea

**Keywords:** aging, circadian rhythm, liver, Nr1d1, Nr1d2, sex difference

## Abstract

Background: Circadian rhythm is associated with the aging process and sex differences; however, how age and sex can change circadian regulation systems remains unclear. Thus, we aimed to evaluate age- and sex-related changes in gene expression and identify sex-specific target molecules that can regulate aging. Methods: Rat livers were categorized into four groups, namely, young male, old male, young female, and old female, and the expression of several genes involved in the regulation of the circadian rhythm was confirmed by in silico and in vitro studies. Results: Gene Ontology and the Kyoto Encyclopedia of Genes and Genomes pathway enrichment analyses showed that the expression of genes related to circadian rhythms changed more in males than in females during liver aging. In addition, differentially expressed gene analysis and quantitative real-time polymerase chain reaction/western blotting analysis revealed that *Nr1d1 and Nr1d2* expression was upregulated in males during liver aging. Furthermore, the expression of other circadian genes, such as *Arntl, Clock, Cry1/2, Per1/2,* and *Rora/c*, decreased in males during liver aging; however, these genes showed various gene expression patterns in females during liver aging. Conclusions: Age-related elevation of Nr1d1/2 downregulates the expression of other circadian genes in males, but not females, during liver aging. Consequently, age-related upregulation of Nr1d1/2 may play a more crucial role in the change in circadian rhythms in males than in females during liver aging.

## 1. Introduction

Aging is associated with a decline and molecular changes in circadian rhythms via age-associated pathways, which aggravate the aging process [1,2,3]. In the literature, transcriptomic data confirmed changes in circadian gene expression in various organs of several aged species [4]. In addition, the disruption and dysfunction of circadian rhythms cause unbalanced homeostasis and age-associated diseases [5,6]. Therefore, malfunctions in circadian rhythms can adversely affect human health and lifespan, indicating that investigations of the circadian system in the aging process are necessary to promote healthy aging and longevity [7,8,9]. These studies suggest that understanding the role of the circadian rhythm in the aging process is important.

Sex has been reported to be one of the factors on the modulation of the circadian rhythm [10,11], which indicates that sex differences are related to differences in sleep patterns, circadian timing, and physiology [12,13]. It is well known that light and food intake are the major cues that entrain circadian rhythm [14,15,16,17,18]. Moreover, sex is associated with changes in the circadian system when exposed to light and food intake [12,19,20,21,22]. In addition, sex differences in circadian rhythms contribute to the regulation of other biological pathways. For example, sex differences in circadian gene expression cause the infection response and inflammation in males and females to differ [23,24]. Furthermore, circadian genes were reported to be involved in metabolic regulation in a sex-different manner [25]. These studies indicate that an understanding of sex differences in circadian rhythms is essential to alleviate various age-related problems; however, the results of studies on sex differences associated with age-related changes in the circadian rhythms remain inconclusive.

The regulation of circadian rhythms has been demonstrated. In mammals, aryl hydrocarbon receptor nuclear translocators, such as (Arntl or Bmal1) and the clock circadian regulator (Clock), organize heterodimers to promote the expression of cryptochrome circadian regulators (*Cry*), period circadian regulators (*Per*), and other circadian genes [26,27]. Cry and Per also organize heterodimers and repress the Arntl–Clock complex, which serves as a negative feedback loop in circadian rhythms [28,29]. In addition, nuclear receptor subfamily 1 group D member 1 (Nr1d1), nuclear receptor subfamily 1 group D member 2 (Nr1d2), RAR-related orphan receptor A (Rora), and RAR-related orphan receptor C (Rorc) are transcription factors that bind retinoic acid-related orphan receptor binding elements (ROREs); however, Nr1d1 and Nr1d2 serve as repressors of ROREs and inhibit the expression of Arntl and Clock, thereby establishing another negative feedback loop in the circadian rhythm. Rora and Rorc form a positive feedback loop to induce the expression of Arntl and Clock [30,31,32]. These studies have demonstrated that circadian genes such as *Nr1d1/2*, *Arntl*, *Clock*, *Cry 1/2*, *Per1/2*, and *Rora/c* construct regulatory systems with several feedback loops; however, how age and sex can change these circadian modulation systems remains unclear.

Therefore, in this study, we analyzed transcriptomic data of rat livers to examine age- and sex-differential expression of the genes and pathways using a systems-biological methodology. Next, we identified expression changes in the age- and sex-differential genes Nr1d1 and Nr1d2, which are involved in the circadian rhythm of male and female rats during liver aging. Finally, we validated the sex-specific expression of Nr1d1/2 and other circadian genes during liver aging.

## 2. Results

### 2.1. Age- and Sex-Related Changes in Gene Expression in Rat Liver

**Figure 1:**
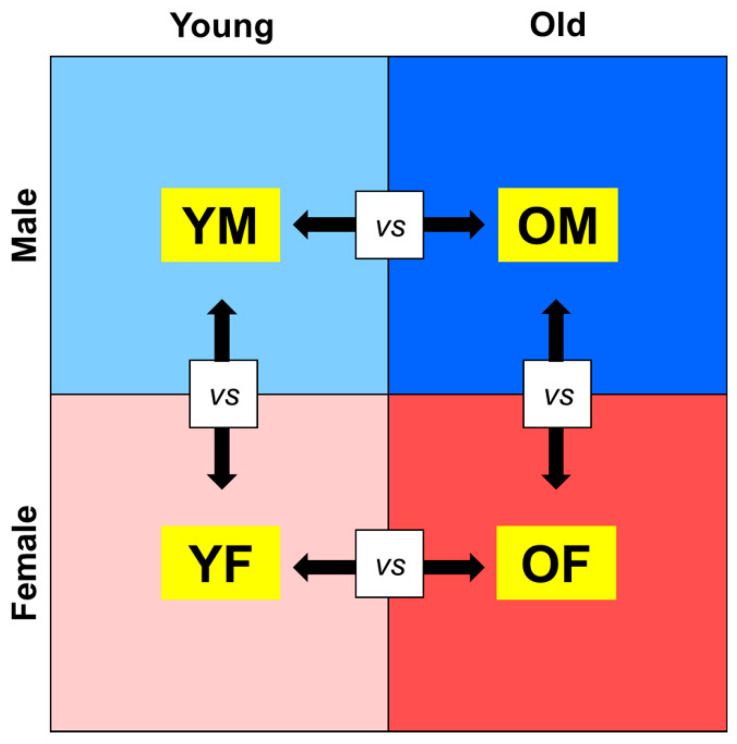
Study designs for transcriptomic analysis of age and sex differences. RNA-Seq data of rat liver tissues were categorized into four groups, and DEGs were calculated by two groups. OM, old male; YM, young male; OF, old female; YF, young female.

**Figure 2:**
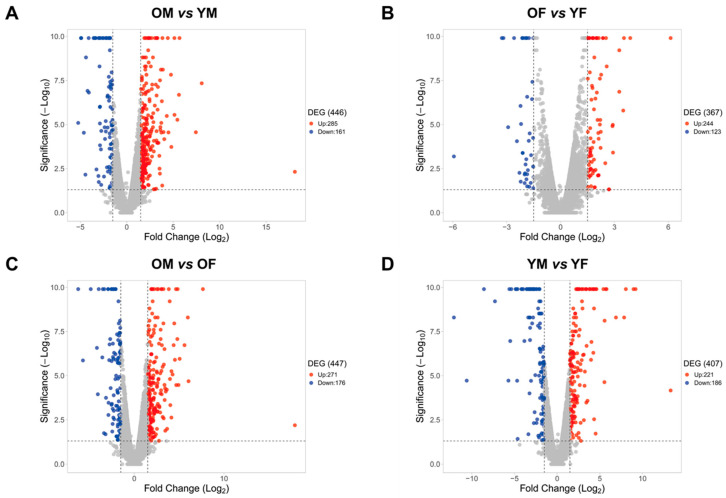
Age- and sex-differentially expressed genes from RNA-Seq data of (**A**) OM vs. YM, (**B**) OF vs. YF, (**C**) OM vs. OF, and (**D**) YM vs. YF from SD rats (*n* = 3 in each group). Red dots represent upregulated genes between two groups. Blue dots represent downregulated genes between two groups. Grey dots represent genes that showed no change between two groups. The criteria for a DEG are |FC| > 1.5 and *p*-value < 0.05. OM, old male; YM, young male; OF, old female; YF, young female.

To determine the differences in gene expression according to age and sex in the liver, we separated RNA-Seq data from the liver tissues of rats into four groups: YM, OM, YF, and OF (young: aged 5 months, old: aged 20 months). Furthermore, DEGs were calculated after comparing the RNA-Seq data of the groups (OM vs. YM, OF vs. YF, OM vs. OF, and YM vs. YF) (Figure 1). We identified 285 upregulated genes and 161 downregulated genes in OM compared with YM, and 244 upregulated genes and 123 downregulated genes were identified in OF compared with YF. Furthermore, we identified 271 upregulated genes and 176 downregulated genes that were altered in OM compared with OF, and 221 upregulated genes and 186 downregulated genes were identified in YM compared with YF (Figure 2). Our data revealed age- and sex-related changes in gene expression during liver aging.

### 2.2. Biological Functions of Genes Whose Expression Was Changed According to Age and Sex in Rat Livers, as Detected by GO Enrichment Analysis

**Figure 3:**
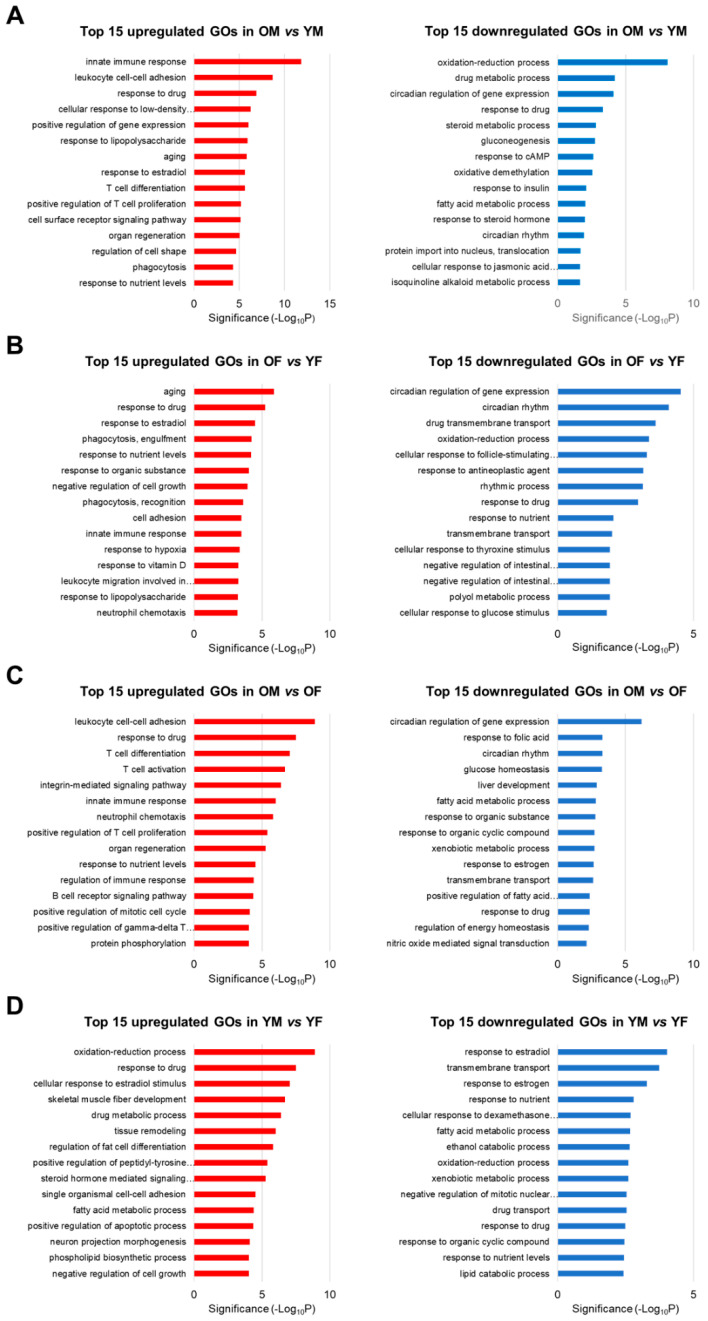
Top 15 Gene Ontology (GO) enrichment analysis of RNA-Seq data of (**A**) OM vs. YM, (**B**) OF vs. YF, (**C**) OM vs. OF, and (**D**) YM vs. YF from SD rats (each *n* = 3). Upregulated GOs of immune response and downregulated GOs of metabolism and circadian rhythm were detected during aging in both sexes. OM, old male; YM, young male; OF, old female; YF, young female.

To identify the biological functions of genes whose expression changed according to age and sex, we used The Database for Annotation, Visualization, and Integrated Discovery (DAVID) to perform a GO enrichment analysis of the DEGs for each dataset that was identified in the results of the DEG calculation. Furthermore, we listed the top 15 GOs for each dataset in Figure 3. As a result, upregulated genes in OM vs. YM were involved in 178 significantly enriched GO terms, including immune response and T cell proliferation. Downregulated genes in OM vs. YM were involved in 31 significantly enriched GO terms, including the circadian regulation of gene expression, circadian rhythms, and the oxidation-reduction process (Figure 3A). In addition, upregulated genes in OF vs. YF were involved in 77 significantly enriched GO terms, including innate immune response and leukocyte migration, and downregulated genes in OF vs. YF were involved in 37 significantly enriched GO terms, which included the circadian regulation of gene expression, circadian rhythms, and the oxidation-reduction process (Figure 3B). In addition, upregulated genes in OM vs. OF were involved in 134 significantly enriched GO terms, including immune response and T cell differentiation/proliferation/activation. Downregulated genes in OM vs. OF were involved in 37 significantly enriched GO terms, which included the circadian regulation of gene expression, circadian rhythms, and various metabolic processes (Figure 3C). In YM vs. YF, no significant sex differences were observed in age-related gene expression, indicating that the genes involved in metabolic processes were differentially expressed in YM and YF (Figure 3D). Notably, OM and OF showed an age-related downregulation of circadian rhythm and the circadian regulation of gene expression. Furthermore, OM showed a substantial reduction in circadian rhythm and the circadian regulation of gene expression compared with OF. These data collectively indicate that males and females showed an elevation in immune response as well as a reduction in metabolism and circadian rhythm during liver aging. We focused on circadian-related GOs, which showed more significant age-related downregulation in OM than in OF.

### 2.3. Biological Pathways of Genes Whose Expression Was Changed According to Age and Sex in Rat Liver, as Detected by KEGG Enrichment Analysis

To confirm the biological pathways of genes whose expressions changed according to age and sex, we performed KEGG enrichment analyses of the DEGs for each dataset that was identified in the results of the previous analysis and then listed pathways in which the DEGs were involved (Table 1). Inflammatory pathways, including the Jak-STAT signaling pathway and cytokine–cytokine receptor interactions, were upregulated in OM vs. YM, while metabolic pathways and circadian rhythms were downregulated in OM vs. YM. In addition, circadian rhythms were downregulated in OF vs. YF, although the metabolisms showed little age-related differences because the genes related to metabolic pathways were upregulated or downregulated, which indicated that metabolic genes were not changed in an age-specific manner. By contrast, immune pathways, including the T cell receptor signaling pathway and B cell receptor signaling pathway, were upregulated in OM vs. OF, while various metabolic pathways, drug metabolism, and circadian rhythms were downregulated in OM vs. OF. Finally, in YM vs. YF, both upregulated and downregulated pathways were involved in various metabolic processes, indicating that YM and YF showed no significant differences in immunity/inflammation and metabolic/circadian gene expression. These results showed that immune and inflammatory pathways were upregulated, while metabolic and circadian pathways were downregulated, in OM compared with OF. Furthermore, as in the GO analysis, KEGG enrichment analyses revealed that circadian rhythms were downregulated in both older males and females; however, these differences were more pronounced in older males than in older females. These results suggest that the circadian rhythm may play a crucial role in the liver aging of males but not females.

### 2.4. Gene Expression of Circadian Genes That Were Differentially Expressed According to Age and Sex in Rat Livers, as Detected by Transcriptomic Analysis

GO and KEGG analyses revealed that the expression of circadian rhythms showed sex differences during aging. Therefore, we selected genes related to the regulation of the circadian rhythm and confirmed their gene expression changes from each dataset. As shown in Table 2, *Nr1d1* and *Nr1d2* were upregulated in OM compared with YM, and their expression was downregulated in OF compared with YF. Conversely, the expression level of *Arntl* (or *Bmal1*) and *Clock* (or *Npas2*) decreased in males during aging, and the expression level of these genes was elevated in females during aging. In addition, *Cry1, Rora,* and *Rorc* showed age-related decreases only in males. By contrast, *Per2* expression was downregulated in both sexes during aging. Furthermore, *Cry2* and *Per1* showed no age-related differences between men and women. These results reveal that several circadian genes, such as *Nr1d1, Nr1d2, Arntl*, and *Clock*, showed sex-specific changes in gene expression during liver aging, which resulted in the differential expression of circadian genes between OM and OF.

### 2.5. Gene Expression of Circadian Genes That Were Differentially Expressed According to Age and Sex in Rat Livers, as Determined via qRT-PCR and Western Blotting

**Figure 4:**
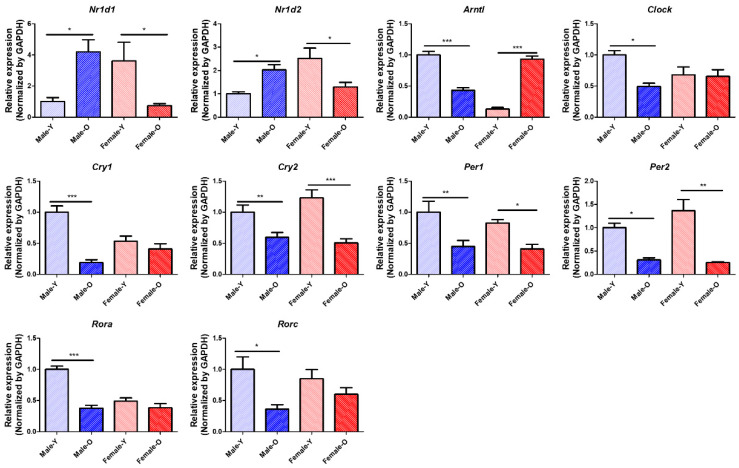
Relative mRNA expression of circadian genes differentially expressed across sex and age in SD rats (*n* = 6 per group). *Nr1d1*, *Nr1d2*, *Arntl*, *Clock*, *Cry1*, *Cry2*, *Per1*, *Per2*, *Rora*, and *Rorc* showed sex differences in their gene expression during liver aging. *Nr1d1*, nuclear receptor subfamily 1 group D member 1; *Nr1d2*, nuclear receptor subfamily 1 group D member 2; *Arntl*, aryl hydrocarbon receptor nuclear translocator like; *Clock*, clock circadian regulator; *Cry1*, cryptochrome circadian regulator 1; *Cry2*, cryptochrome circadian regulator 2; *Per1*, period circadian regulator 1; *Per2*, period circadian regulator 1; *Rora*, RAR-related orphan receptor A; *Rorc*, RAR-related orphan receptor C; Y, young; O, old; * *p* < 0.05, ** *p* < 0.01, and *** *p* < 0.001 between the two groups.

**Figure 5:**
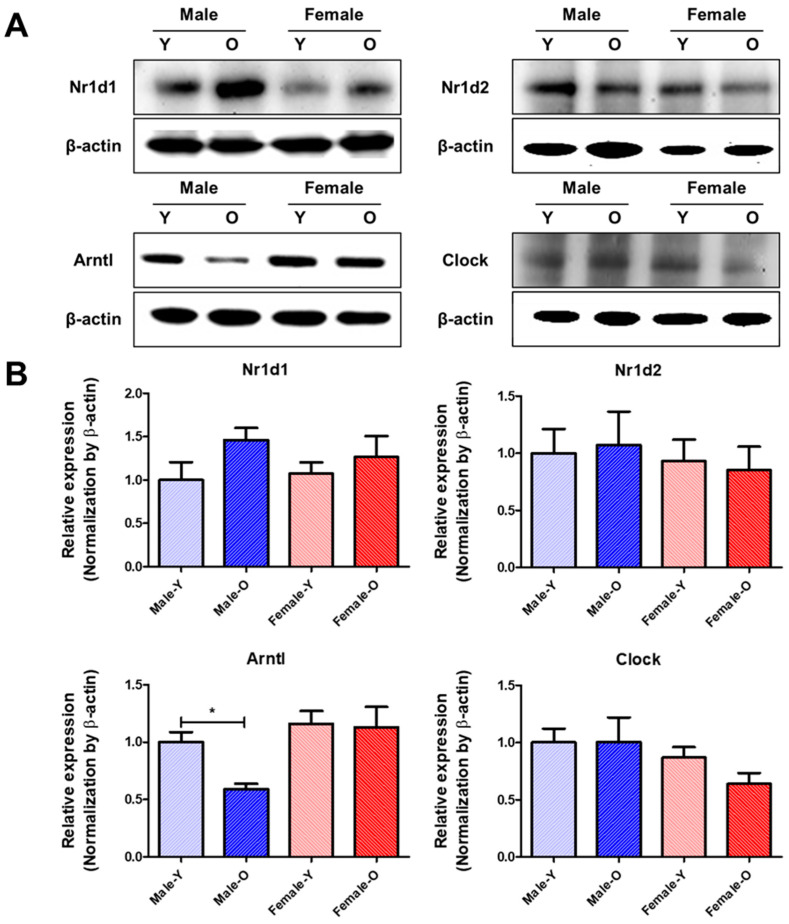
Protein expressions of Nr1d1/2, Arntl, and Clock which were differentially expressed according to sex and age in SD rats (*n* = 4 or 6 per group). (**A**) Representative band images of Nr1d1/2, Arntl, and Clock. (**B**) Relative expression of Nr1d1/2, Arntl, and Clock. These proteins showed sex differences in their expression during liver aging. Nr1d1, nuclear receptor subfamily 1 group D member 1; Nr1d2, nuclear receptor subfamily 1 group D member 2; Arntl, aryl hydrocarbon receptor nuclear translocator like; Clock, clock circadian regulator; Y, young; O, old. * *p* < 0.05 between the two groups.

Transcriptomic analysis revealed that several circadian genes, such as *Nr1d1, Nr1d2, Arntl,* and *Clock*, show sex differences in gene expression during the aging process. qRT-PCR was performed to validate the expression of the circadian genes (Figure 4). The expression of *Nr1d1* and *Nr1d2* was significantly increased in males and decreased in females during aging. Conversely, *Arntl* was significantly downregulated in males and upregulated in females during the aging process. *Clock* expression levels also significantly decreased in OM compared with YM, and there was no age-related change in females. In addition, *Cry2, Per1,* and *Per2* were significantly downregulated in both sexes during aging. *Cry1, Rora,* and *Rorc* were significantly downregulated in old males, and the genes showed no gene expression changes in old females. Furthermore, western blotting was performed to detect the levels of Nr1d1, Nr1d2, Arntl, and Clock (Figure 5). The protein levels of Nr1d1 and Nr1d2 showed a tendency to be higher in males during aging. On the other hand, the protein level of Arntl was lower in old males than in young males, while its protein level was not changed in females during the aging process. In addition, the protein level of Clock was not changed in both sexes during aging. These results indicate that *Nr1d1* and *Nr1d2* were upregulated in old males, which downregulated the protein expression of their target gene, *Arntl* in aged males. Although Nr1d1/2 were not significantly overexpressed in protein levels, their protein expression and activity may be involved in the regulation of Arntl. Collectively, our study suggested that upregulation of Nr1d1 and Nr1d2 may regulate other circadian genes such as *Arntl* and *Clock* to a greater degree in males than in females during liver aging. These results highlight that sex differences can affect the expression of circadian genes during the aging of the liver.

## 3. Discussion

In this study, we conducted a transcriptomic analysis of RNA-Seq data from rat livers, which revealed that the DEGs, GOs, and KEGG pathways related to inflammation were upregulated and that those related to metabolism were downregulated during aging. Notably, DEGs, GOs, and KEGG pathways related to circadian rhythms were altered in both sexes during the aging process. In addition, transcriptomic and experimental analyses also showed that the expressions of circadian genes *Arntl*, *Clock*, *Cry1/2*, *Per1/2*, and *Rora/c* were downregulated in males and that the expression level of *Nr1d1/2* was higher in males than in females during liver aging.

Circadian rhythms interact with inflammatory pathways, and disruption induces inflammation and inflammatory diseases [33,34]. In addition, inflammation disturbs the circadian rhythm and contributes to a vicious cycle of circadian rhythm disruption and inflammation [35]. Furthermore, circadian genes were demonstrated to be related to the immune system and further regulate the expression of various cytokines, chemokines, and immune-related genes [36,37]. Our study also agrees with these studies and suggests that downregulation of circadian genes in males can cause male-specific disruptions of the circadian rhythm and worsen inflammatory responses in males. Therefore, further research is necessary to discover inflammatory transcription factors that affect the expression of circadian genes and confirm the sex-specific changes in circadian genes under inflammatory conditions, such as LPS stimulation.

The literature has reported that the circadian rhythm interacts with various metabolic proteins and systemically regulates various metabolic pathways [38,39,40]. In addition, disruption of the circadian rhythm can cause several metabolic problems such as obesity, insulin resistance, chronic inflammation, and hepatic diseases [41,42,43]. These results suggest the importance of circadian rhythms, which are involved in the regulation of the metabolism. Our study also showed that genes related to several metabolic pathways were downregulated, especially in males, during aging. Therefore, we suggest that the male-specific disruption of circadian genes may be related to the male-specific downregulation of metabolic genes.

Nr1d1 regulated the expression of cytokines under LPS stimulation [44]. Nr1d1 has been reported to alleviate inflammation during inflammatory diseases such as rheumatoid arthritis and hepatitis [45,46]. Furthermore, Nr1d1 alleviated inflammation under LPS conditions by regulating inflammatory pathways such as the NF-κB signaling pathway [47,48]. Nr1d1 also affects immunity via interactions with molecules and pathways associated with immune response [46,49]. In addition, Nr1d1/2 regulated metabolic functions including steroid metabolism [31,50,51,52]. Other studies have suggested that elevated Nr1d1 expression may improve metabolism [32,53]. Furthermore, activating Nr1d1/2 with agonists has been demonstrated to result in anti-inflammatory effects, the regulation of metabolism, and improvements in inflammatory and metabolic diseases [44,45,54,55]. Our study revealed that Nr1d1/2 were overexpressed in aged male livers. This finding indicates that these genes may regulate inflammatory and metabolic changes to maintain liver homeostasis. However, this compensation response could disrupt circadian rhythms because chronic or irregularly high expressions of Nr1d1/2 may suppress the expression of other circadian genes, which is consistent with our results. Therefore, molecular mechanisms regulating Nr1d1/2 are necessary to study the male-specific increase in inflammation and deterioration of the metabolism during the aging process.

Except for *Nr1d1/2*, our data demonstrated that the expression of circadian genes decreased in males during the aging process. Aging induces dysfunction in Arntl and Clock, which worsens the aging process and metabolic diseases [56,57,58]. In addition, the suppression of cellular senescence and inflammatory diseases that are aggravated by Cry1/2 has been demonstrated [59,60]. Furthermore, the expression level of Per1/2 decreases during aging, and the downregulation of *Per1/2* contributes to cytokine production and inflammatory disease pathogenesis [61,62,63]. Studies have indicated that the repression of Rora enhances inflammation and cytokine production and contributes to the deterioration of the metabolism [64,65,66]. Although the role of Rorc, as to whether the protein has proinflammatory or anti-inflammatory effects [67,68,69], remains unclear. It is clear that molecules targeting Rora/c can be effective agents to regulate inflammation- and metabolism-related diseases [70,71,72,73,74]. Furthermore, Nr1d1/2 and Rora/c compete to bind to ROREs; therefore, modulation of Rora/c may play a key role in regulating Nr1d1/2 activation [75].

Our study revealed that the circadian rhythm is affected by sex differences. The effects of hormones related to circadian rhythm occur in a sex-dependent manner [76]. In addition, sex hormones have been reported to regulate circadian rhythms and behavior [77]. Furthermore, Nr1d1 is affected by sex hormones. For example, testosterone inhibits Nr1d1 expression, and Nr1d1 is involved in the inhibition of testosterone synthesis [78,79]. By contrast, progesterone and estradiol induce the expression of Nr1d1, and Nr1d1 inhibits estradiol synthesis [80,81]. These studies indicate that a decrease in testosterone during male aging induces Nr1d1 expression in OM, and a decrease in female sex hormones during female aging alleviates Nr1d1 expression in old females, which corresponds with our results. Furthermore, upregulated Nr1d1 may deepen aging in males because it can inhibit testosterone synthesis. In addition, calorie restriction, an anti-aging strategy, affects the expression of circadian genes, including *Nr1d1* and *Rorc*, in a sex-dependent manner, which proves the presence of sex differences in circadian rhythms during aging [82]. Additionally, the disruption of *Rora* expression has been reported to be more critical in males than females because Rora was highly correlated with its target genes in males [83]. These studies suggest that males may exhibit greater changes in circadian rhythm during liver aging, which supports our data that showed a more significant downregulation in OM than in OF.

## 4. Materials and Methods

### 4.1. Animals

Six young male rats (YM; 5 months old), six old male rats (OM; 20 months old), six young female rats (YF; 5 months old), and six old female rats (OF; 20 months old) were purchased from Samtako [84,85,86,87]. All Sprague Dawley (SD) rats were maintained at 23 ± 2 °C with a relative humidity of 60 ± 5% and a 12 h light and dark cycle. The rats were fed ad libitum with a water vehicle and a normal chow diet purchased from Biopia (Gunpo, Gyeonggi-do, Korea). Their tissues were immediately frozen in liquid nitrogen for isolation and analysis. All animal experiments were approved by the Pusan National University Institutional Animal Care and Use Committee. This study complied with all animal testing guidelines (approval number PNU-2019-2282) issued by the Pusan National University.

### 4.2. RNA Sequencing (RNA-Seq)

The total RNA was isolated from the liver samples using TRIzol reagent (Invitrogen, Carlsbad, CA, USA), and samples from each group were pooled in equal quantities for RNA-Seq (*n* = 3 in each group). RNA integrity was assessed using an Agilent 2100 BioAnalyzer and an Infinite F200 (concentration value greater than 65 ng/µL, quantity value greater than 1 µg, and RNA integrity number value greater than 6). Libraries were prepared for 150-bp paired-end sequencing using the TruSeq stranded mRNA Sample Preparation Kit (Illumina, CA, USA). mRNA molecules were purified and fragmented from 1 μg of the total RNA using oligo (dT) magnetic beads. Fragmented mRNAs were synthesized as single-stranded cDNAs through random hexamer priming. Double-stranded cDNA was prepared by applying it as a template for second strand synthesis. After the sequential process of end repair, A-tailing, and adapter ligation, cDNA libraries were amplified with a polymerase chain reaction (PCR). The quality of the cDNA libraries was evaluated using an Agilent 2100 BioAnalyzer (Agilent, Santa Clara, CA, USA). They were measured using the KAPA library quantification kit (Kapa Biosystems, Wilmington, MA, USA) according to the manufacturer’s library quantification protocol. After the cluster amplification of denatured templates was performed, paired-end sequencing (2 × 150 bp) was performed using Illumina NovaSeq6000 (Illumina, San Diego, CA, USA).

### 4.3. Differential Expressed Gene (DEG) Analysis

The adapter sequences and any ends of the reads with a Phred quality score less than 20 were trimmed and reads shorter than 50 bp were removed simultaneously using cutadapt v.2.8 [88]. Filtered reads were mapped to the reference genome related to the species using the aligner STAR v.2.7.1a, by applying ENCODE standard options (refer to “Alignment” in the “Help” section in the html report) with the “-quant Mode Transcriptome SAM” option for estimating the transcriptome expression level [89]. Gene expression estimation was performed using RSEM v.1.3.1, considering the direction of the reads that corresponded to the library protocol using the “--strandedness” option. To improve the accuracy of the measurement, we applied the “--estimate-rspd” option [90]. All other options were set to default values. FPKM and TPM values were calculated to normalize the sequencing depth among the samples. Based on the estimated read counts in the previous step, DEGs were identified using R package TCC v.1.26.0 [91]. The TCC package applies robust normalization strategies to compare tag count data. The normalization factors were calculated using the iterative edgeR method [92]. After calculation, we selected DEGs whose expression was changed by 1.5-fold or more and whose *p* value was less than 0.05. DEGs are presented in Table S1. A volcano plot of the DEGs for each dataset was constructed using the criteria, which are based on the VolcaNoseR website [93].

### 4.4. Enrichment Analysis of the DEGs

Gene Ontology (GO) enrichment analysis was used to analyze the biological functions of the genes, and the Kyoto Encyclopedia of Genes and Genomes (KEGG) pathway enrichment analysis was performed to investigate the signaling pathways related to the unique DEGs. The Database for Annotation, Visualization, and Integrated Discovery (DAVID) was used to perform GO and KEGG enrichment analyses (filtering options: *p* < 0.05). The results of the GO and KEGG enrichment analyses are listed in Tables S2 and S3. For the overlapping KEGG pathways, we overlapped the enriched KEGG pathways from the datasets and identified common KEGG pathways that were consistently altered in the datasets.

### 4.5. Quantitative Real-Time Polymerase Chain Reaction (qRT-PCR)

Primers for qRT-PCR were synthesized by Bioneer, Inc. (Daejeon, Korea). Total RNA was isolated from rat liver tissues (20 mg) using TRIzol reagent (Invitrogen, Carlsbad, CA, USA) (*n* = 6 per group) and was reverse-transcribed using the cDNA synthesis kit from GenDEPOT. qRT-PCR analysis was performed to quantify mRNA levels using SYBR Green (Bioneer, Daejeon, Korea) and the CFX Connect System (Bio-Rad Laboratories Inc., Hercules, CA, USA). Primer sequences are listed in Table S4.

### 4.6. Cytosolic Protein Extraction from Liver Tissues

Frozen liver tissues (150–200 mg) were ground using liquid nitrogen in a mortar and pestle. Ground liver tissues were homogenized in 1 mL hypotonic lysis buffer. Buffer A was composed of KCl (10 mM), MgCl2 (2 mM), dithiothreitol (DTT) (1 mM), EDTA (0.1 mM), PMSF (0.1 mM), pepstatin (1 μM), leupeptin (2 μM), β-glycerophosphate (20 mM), NaF (20 mM), Na3VO4 (2 mM), and 4-(2-hydroxyethyl)-1-piperazineethanesulfonic acid (HEPES), pH 7.4 (10 mM). The tissue homogenizer was used for 20 s. After homogenates were incubated on ice for 15 min, 125 μL of 10% Nonidet P-40 (NP-40) was added to the homogenates and mixed for 15 s, and the mixture was centrifuged at 14,000× *g* for 2 min at 4 °C. The supernatants were deemed cytosolic fractions.

### 4.7. Western Blotting

Total protein extraction samples of the rat liver tissues were boiled for 5 min in a loading buffer containing 0.2% bromophenol blue, 125 mM Tris-HCl, 10% 2-mercaptoethanol, pH 6.8, and 4% sodium dodecyl sulfate. Equal amounts of protein (8–10 μg) were loaded and separated via SDS-PAGE using 10% gels and then transferred to PVDF membranes at 25 V for 10 min using a semidry transfer method. Protein-transferred membranes were soaked in 5% nonfat milk buffer containing 100 mM NaCl, 10 mM Tris (pH 7.5), and 0.1% Tween-20 for 2 h. The membranes were washed four times in TBS-Tween buffer for 8 min and were immunoblotted with specific primary antibodies (1:1000) dilution at 4 °C overnight. This was followed by HRP-conjugated secondary incubation (1:10,000 dilution) according to the manufacturer’s instructions of each primary antibody for 1 h at RT. Antibody labeling was detected using enhanced chemiluminescence in accordance with the manufacturer’s instructions. Molecular weights were determined using wide-range protein markers. The resulting immunoblots were visualized using Chemiluminescent HRP Substrate (Advansta, San Jose, CA, USA), Davinchchemi CAS-400 (Davinch-K, Seoul, Korea), and ImageJ software (NIH, Bethesda, MD, USA), according to the manufacturers’ instructions. Antibodies against Nr1d1 (sc-100910), Nr1d2 (sc-398252), Arntl (sc-365645), and β-Actin (sc-47778) were purchased from Santa Cruz (Santa Cruz Biotechnology, Santa Cruz, CA, USA). Clock antibody (#5157) was obtained from Cell Signaling Technology (Danvers, MA, USA), and secondary antibodies (GTX213110-01, GTX213111-01) were purchased from Genetax (Irvine, CA, USA).

### 4.8. Statistical Analysis

The Student’s *t*-test was used to analyze the differences between the two groups. Statistical significance was set at *p* < 0.05. Statistical analyses were performed using GraphPad Prism 5 (La Jolla, CA, USA).

## 5. Conclusions

**Figure 6:**
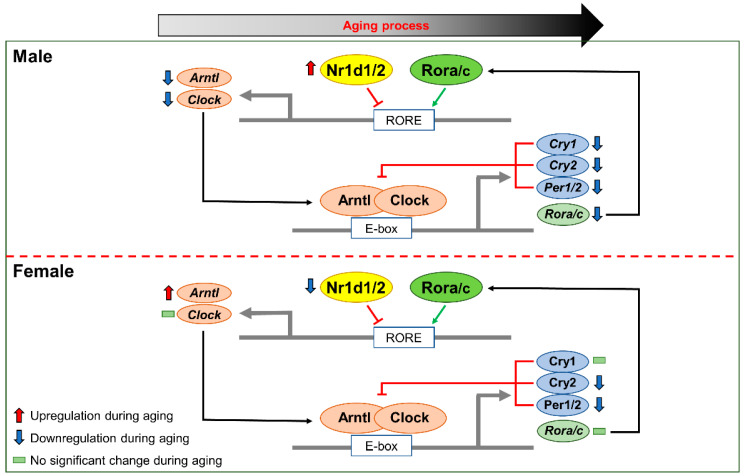
Changes in the expression of circadian genes due to sex-different expression of Nr1d1/2. During the liver aging process, Nr1d1/2 upregulation suppressed expression of *Arntl* and *Clock* and resulted in the downregulation of other circadian genes, such as *Cry1/2, Per1/2,* and *Rora/c.* Male-specific upregulation of Nr1d1/2 might be involved in the change in circadian rhythm during male liver aging.

In conclusion, via transcriptomic analysis, this study revealed that the genes involved in most circadian rhythms were downregulated in males, but not in females, during liver aging. In detail, *Nr1d1/2* expressions were upregulated in males and downregulated in females, leading to sex-different disruptions of the negative feedback loop in the circadian rhythm via regulation of their target genes, such as *Arntl* and *Clock*, during liver aging. Therefore, upregulation of *Nr1d1/2* expression may play an important role in disrupting the negative feedback loop in the circadian rhythm of sex-specific male rats during the liver aging process (Figure 6). Our analysis highlighted that male-specific upregulated genes *Nr1d1* and *Nr1d2* play an important role in the circadian rhythm of male rats, but not in female rats, in the liver aging process.

## Figures and Tables

**Table 1:** KEGG pathways of the DEGs involved in immunity, inflammation, metabolism, and circadian rhythm from RNA-Seq data in OM vs. YM, OF vs. YF, OM vs. OF, and YM vs. YF.

Dataset	Up	Down
OM vs. YM	Natural killer cell-mediated cytotoxicity, Hematopoietic cell lineage, Rheumatoid arthritis, Cell adhesion molecules (CAMs), Fc epsilon RI signaling pathway, Antigen processing and presentation, Asthma, Jak-STAT signaling pathway, Cytokine—cytokine receptor interaction, Leukocyte transendothelial migration, Inflammatory bowel disease (IBD), B cell receptor signaling pathway, Type I diabetes mellitus, T cell receptor signaling pathway, Retinol metabolism, Primary immunodeficiency, Central carbon metabolism in cancer	Steroid hormone biosynthesis, Metabolic pathways, Retinol metabolism, Linoleic acid metabolism, Drug metabolism—other enzymes, Circadian rhythm
OF vs. YF	Maturity onset diabetes of the young, CAMs, Glycine, serine and threonine metabolism, Metabolic pathways	Drug metabolism—cytochrome P450, Nicotinate and nicotinamide metabolism, Glutathione metabolism, Metabolism of xenobiotics by cytochrome P450, Metabolic pathways, Pentose and glucuronate interconversions, Circadian rhythm, Steroid hormone biosynthesis, Galactose metabolism, Retinol metabolism, Fructose and mannose metabolism, Tyrosine metabolism
OM vs. OF	Natural killer cell-mediated cytotoxicity, CAMs, Rheumatoid arthritis, Leukocyte transendothelial migration, Hematopoietic cell lineage, Fc epsilon RI signaling pathway, T cell receptor signaling pathway, Primary immunodeficiency, Antigen processing and presentation, Intestinal immune network for IgA production, Asthma, Inflammatory bowel disease (IBD), B cell receptor signaling pathway	Retinol metabolism, Circadian rhythm, Ascorbate and aldarate metabolism, Fatty acid elongation, Biosynthesis of unsaturated fatty acids, Metabolic pathways, Metabolism of xenobiotics by cytochrome P450, Drug metabolism—cytochrome P450, Steroid hormone biosynthesis, Drug metabolism—other enzymes, Histidine metabolism, Pentose and glucuronate interconversions, PPAR signaling pathway
YM vs. YF	Steroid hormone biosynthesis, Metabolic pathways, PPAR signaling pathway, Choline metabolism in cancer	Drug metabolism—cytochrome P450, Steroid hormone biosynthesis, Retinol metabolism, Butanoate metabolism, Metabolic pathways, Metabolism of xenobiotics by cytochrome P450, Drug metabolism—other enzymes, Ascorbate and aldarate metabolism, Biosynthesis of unsaturated fatty acids, Fatty acid elongation

**Table 2:** Fold change in genes related to circadian rhythm.

Gene	FC (OM vs. YM)	FC (OF vs. YF)	FC (OM vs. OF)
*Nr1d1*	6.680703355	−2.056227653	3.680750602
*Nr1d2*	2.602683711	−2.345669898	1.607701981
*Arntl (or Bmal1)*	−2.114036081	3.972369982	−1.969732886
*Clock (or Npas2)*	−1.859609885	29.65081798	−2.602683711
*Cry1*	−3.226567037	1.155886707	−2.411615655
*Cry2*	−1.209994089	−1.317679952	−1.112650121
*Per1*	−1.441928871	−1.009471374	−1.783857039
*Per2*	−2.042024251	−2.02791896	−1.822602561
*Rora*	−2.789487333	1.340712592	−2.462288827
*Rorc*	−1.821339667	1.134455485	−1.876442393

The color codes include red for highly upregulated genes; pink for slightly upregulated genes; blue for highly downregulated genes; light blue for slightly downregulated genes. Fold change in bold means that the genes were differentially expressed in each dataset.

## Data Availability

The data presented in this study are available on request from the corresponding author. The data are not publicly available due to privacy.

## Supplementary Materials

The following supporting information can be downloaded at: https://www.mdpi.com/article/10.3390/ijms231710032/s1.
